# Toll-Like Receptors and the Response to Radiotherapy in Solid Tumors: Challenges and Opportunities

**DOI:** 10.3390/vaccines11040818

**Published:** 2023-04-07

**Authors:** Ryma Haroun, Sahar Naasri, Ayman J. Oweida

**Affiliations:** Department of Nuclear Medicine and Radiobiology, Faculty of Medicine and Health Sciences, Université de Sherbrooke, Sherbrooke, QC J1N 0Y8, Canada

**Keywords:** cancer, radiotherapy, toll-like receptors, innate immunity, adaptive immunity, immunotherapy

## Abstract

Toll-like receptors (TLRs) are indispensable for the activation, maintenance and halting of immune responses. TLRs can mediate inflammation by recognizing molecular patterns in microbes (pathogen-associated molecular patterns: PAMPs) and endogenous ligands (danger-associated molecular patterns: DAMPs) released by injured or dead cells. For this reason, TLR ligands have attracted much attention in recent years in many cancer vaccines, alone or in combination with immunotherapy, chemotherapy and radiotherapy (RT). TLRs have been shown to play controversial roles in cancer, depending on various factors that can mediate tumor progression or apoptosis. Several TLR agonists have reached clinical trials and are being evaluated in combination with standard of care therapies, including RT. Despite their prolific and central role in mediating immune responses, the role of TLRs in cancer, particularly in response to radiation, remains poorly understood. Radiation is recognized as either a direct stimulant of TLR pathways, or indirectly through the damage it causes to target cells that subsequently activate TLRs. These effects can mediate pro-tumoral and anti-tumoral effects depending on various factors such as radiation dose and fractionation, as well as host genomic features. In this review, we examine how TLR signaling affects tumor response to RT, and we provide a framework for the design of TLR-based therapies with RT.

## 1. Introduction

**Figure 1:**
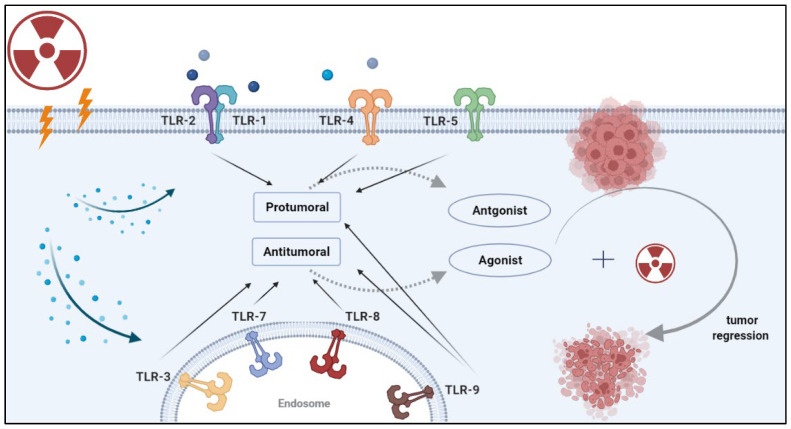
Strategies for combining radiotherapy with TLR-based immunotherapy. Radiation induces the release of damage-associated molecular patterns (DAMPs) by damaged or dying cells. DAMPs can be recognized by pattern recognition receptors (PRRs) such as TLRs. Once DAMPs bind to these receptors, they initiate a signaling cascade that leads to a pro-tumor or anti-tumor effect, depending on the specific TLR and the cancer type. Rationale combinations of TLR-based therapy with RT can enhance tumor recognition by antigen-presenting cells and lead to durable antitumor immunity.

Toll-like receptors (TLRs) are central to the initiation of innate and adaptive immune responses. TLRs belong to the family of pattern recognition receptors (PRRs), which recognize molecular patterns in microbes (PAMPs) and endogenous ligands (DAMPs) [1]. There are 10 known TLRs in humans that recognize microbial ligands and products of damaged tissues [2]. Broadly, TLRs can be expressed on the cell surface or in the endosome. Cell surface TLRs recognize the lipopolysaccharide (LPS) of gram-negative bacteria (TLR4), bacterial lipoproteins (TLRs 1,2,6) and flagellin (TLR5) [3]. Endosomal TLRs mainly recognize nucleic acids, such as double-stranded RNA (TLR3), single-stranded RNA (TLR7) and double-stranded DNA (TLR9). With the exception of TLR3, TLRs activate the myeloid differentiation factor 88 (MyD88) signaling pathway. TLR3 signals in a TIR-domain-containing adapter-inducing interferon-b (TRIF)-dependent manner. TLR4 is the only receptor that can induce both MyD88 and TRIF-dependent signaling pathways. These pathways induce the synthesis or release of pro- and anti-inflammatory cytokines via the activation of transcription factors such as NF-κB, interferon regulatory factor (IRF)-3/7, AP-1 and others [4,5]. Such cytokines are often responsible for the recruitment of intratumoral immune cells and the resulting tumor immune phenotypes [6]. Given their role in mediating immune responses, the role of TLRs in tumor progression and tumor response to therapy remains poorly understood. In particular, the response to radiotherapy involves the induction of various types of cell damage and/or death which can activate TLRs and initiate downstream signaling. In this review, we cover the different roles of TLRs in cancer, and explore the mechanisms by which TLRs can lead to tumor progression or regression. Several prior review papers have focused on clinical aspects related to TLRs in cancer [7,8,9,10]. In addition, a recent clinical trials watch summarized the clinical studies to date that integrate TLRs in the treatment of cancer patients [11]. The reader is referred to those publications for clinical insight into TLR-based treatments. Here, we discuss the mechanistic effects of radiation on the expression of TLRs, and address how radiotherapy and TLR-based treatments can improve the radiotherapy response in solid tumors (Figure 1).

## 2. Activation and Inhibition of TLRs in Cancer

TLRs are expressed in a variety of cells, including T lymphocytes, monocytes, dendritic cells, alveolar epithelial cells, smooth muscle cells and fibroblasts [12]. Tumor cells have also been shown to express TLRs in ways that promote tumor growth, invasion, anti-apoptotic activity and treatment resistance [13,14]. At the time of this review, eight TLRs (TLR 1, 2, 3, 4, 5, 7, 8 and 9) have been found to be expressed in solid cancer cell lines, including lung, breast, head and neck, colorectal and pancreatic [15]. These are briefly reviewed below and summarized in Table 1.

### 2.1. TLR1 and TLR2—Pro and Anti-Tumoral Roles

Although most TLRs act alone, TLR2 forms heterodimers with TLR1 or TLR6, allowing it to recognize a wide range of exogenous or endogenous molecules [12,16]. The expression of functional TLR2 is found in epithelial cells, but it is also expressed in colorectal (CRC), gastric and ovarian tumors [17,18,19,20]. In CRC, TLR2 levels were associated with malignant transformation and lower overall survival (OS) [20]. Upregulation of TLR2 can promote gastric tumorigenesis independently of tumor inflammation [19], while its knockdown has been shown to inhibit the proliferation of CRC tumors [18]. TLR2 was shown to be regulated by miR-143 which, when expressed, suppresses TLR2 activation, resulting in reduced invasion and migration of CRC cells and reduced tumor growth [20]. 

In contrast to these studies showing a pro-tumorigenic role for TLR1/2, Deng et al. showed that TLR1/2 expression is related to a good prognosis in lung cancer [21]. Their in vivo study showed that treatment with bacterial lipoprotein (BLP), a TLR1/2 agonist, decreased tumor growth in the lung LLCI C57BL/6 mouse model compared to control mice. BLP treatment reduced the percentage of myeloid-derived suppressor cells (MDSCs) in the blood, spleen, and tumor tissues. They further showed that BLP treatment stimulates MDSCs conversion into M1-type macrophages [22]. BLP was also shown to have anti-tumorigenic properties in animal models of lung cancer and melanoma, where complete tumor regression and development of a memory response were observed [22]. The anti-tumor effect of activating TLR1/2 was shown to be a consequence of the reciprocal modulation of effector T cells (Teff) and regulatory T cells (Treg). BLP effectively abrogated Treg function by downregulating Foxp3 expression, resulting in a reduction of Tregs cell numbers and diminished suppressive activity in a TLR2-dependent manner. On the other hand, it increased the proliferation and cytotoxicity of tumor-specific Teff cells. A clinical trial integrating a TLR2 agonist with chemotherapy showed a survival benefit in squamous cell carcinoma patients compared to chemotherapy alone [23].

Based on the limited number of cancer studies on TLR1 and TLR2, it appears that their modulation is cancer-type dependent, with a pro-tumoral role in CRC, gastric and ovarian tumors and an anti-tumoral role in melanoma and lung cancer.

### 2.2. TLR3—Mostly Anti-Tumoral Effects Mediated by Dendritic Cells and CD8 T Cells

Like other TLRs, TLR3 is primarily expressed in innate immune cells such as macrophages, dendritic cells (DCs) and natural killer (NK) cells [24]. TLR3 recognizes dsRNA derived from viruses or host RNA. Unlike other TLRs, TLR3 does not participate in the MyD88 signaling pathway, but rather in the TRIF-dependent pathway [5]. Several studies demonstrated that loss of function TLR3 polymorphisms are associated with an increased risk of head and neck squamous cell carcinoma (HNSCC) [25], hepatocellular carcinoma (HCC) [26,27] and CRC [28,29]. High TLR3 expression has been associated with a good prognosis in NSCLC, HCC and neuroblastoma [26,30,31,32]. TLR3 activation in vitro has been shown to induce apoptosis in the lung cancer cell lines Calu-3 (adenocarcinoma) and H460 (large cell carcinoma), and is associated with caspase-3 activation in human adenocarcinoma NSCLC tissue [33]. Moreover, TLR3 expression in cancer cells activated the CD103+ lung dendritic cell subset, which is specifically associated with the processing of antigens derived from apoptotic cells and their presentation to CD8 T cells [34]. TLR3 has also been considered as a favorable prognostic biomarker of lung cancer, either by activating apoptosis or promoting autophagy [35]. The use of a TLR3 agonist enhanced T cell infiltration in lung tissue, which is essential for tumor immunity within the tumor microenvironment (TME) [36,37]. A clinical trial showed that using an advanced form of TLR3 agonist (Poly-ICLC) promotes cancer cell death and inhibits metastasis by activating several cancer suppressors and rebuilding an immunosuppressive tumor microenvironment [38]. In vitro studies showed that TLR3 activation induces apoptosis in human breast cancer cells, melanoma cells and clear cell renal carcinoma cells, as well as in cervical and prostate cancer [39,40]. These studies revealed that apoptosis induced by poly (I:C) is linked to the production of type I IFN. In HCC, the suppression of TLR3 increased tumor proliferation, angiogenesis, and inhibited apoptosis of liver cancer cells [41,42]. 

In contrast to the majority of studies showing an anti-tumorigenic role for TLR3 activation, a few correlative studies have suggested a pro-tumoral role. In esophageal cancer cells, high TLR3 expression was associated with a higher probability of lymph-node metastasis and increased depth of invasion [27]. In breast and gastric cancers, increased TLR3 expression was associated with poor prognosis and worse overall survival [43,44]. In HNSCC, strong TLR3 expression by IHC correlated with poorly differentiated tumors [45]. The same study further analyzed the response of one cell line (OC2) to TLR3 stimulation, and observed an increase in the phosphorylation of IRF3 and IκB and a resulting induction in the secretion of IL6 and CCL5, which are closely related to tumor aggressiveness. Another study by Zhan et al. showed that activation of TLR3 using poly (I:C) induced autophagy in lung cancer cell lines, which in turn induced the expression of the pro-tumorigenic cytokines IL6, CCL2, CCL20 and VEGFA [46]. Both of these functional studies on TLR3 were based on in vitro experiments and may have involved cell lines that depend on TLR3 activation for proliferation, survival and migration.

### 2.3. TLR4—Pro-Tumoral—Cancer Cell Intrinsic Activation

Most TLR4-expressing cells in humans are of myeloid origin. Numerous studies involving TLR4 in cancer have focused on lung cancer. Analysis of NSCLC clinical samples and excised normal lung tissue showed an increase in TLR4 expression in tumor samples compared to normal tissue [47]. TLR4 expression was correlated with FoxP3 expression in these samples [47]. In a similar study, TLR4 expression was positively correlated with tumor differentiation [48]. It has also been shown that TLR4, MyD88 and HMGB1 are highly expressed in invasive breast cancer cell lines compared to non-invasive cell lines [49]. TLR4 signaling was shown to promote immune escape of human lung cancer cells by inducing immunosuppressive cytokines and apoptosis resistance [50]. In addition, TLR4 activation has been shown to promote invasiveness of human breast cancer cells and to be overexpressed in patients with lymph node metastasis [51]. Activation of TLR4 by LPS has been shown to induce resistance of lung cancer cells to TNFα or TRAIL-induced apoptosis through NF-κB upregulation [50]. A consequence of TLR4 ligation is the production of immunosuppressive cytokines such as transforming growth factor-β (TGF-β), vascular endothelial growth factor (VEGF) and the pro-angiogenic chemokine interleukin-8 (IL-8). A recent study showed that the immune checkpoint protein B7 homolog 3 (B7-H3) promotes invasion and metastasis of pancreatic cancer cells by inducing TLR4 expression [52]. The study showed that silencing of TLR4 decreased soluble B7-H3-induced activity of NF-κB and reduced the expression of IL-8 and VEGF in pancreatic cancer cells. Animal experiments further showed that tumor cells with TLR4 knockdown show a decreased ability to metastasize compared with the control tumor cells after being induced with soluble B7-H3. Blocking TLR4 signaling was shown to significantly improve the response to paclitaxel therapy in breast cancer [53,54]. In MDA-MB-231 breast cancer cells, TLR4 silencing reduced cell proliferation and secretion of the proinflammatory cytokines IL8 and IL6, which are known to contribute to immune evasion and resistance to immunotherapy [55,56]. 

### 2.4. TLR5—Anti-Tumoral—Action Primarily Mediated by Neutrophils

TLR5 is expressed constitutively in epithelial cells and immune cells, such as monocytes, neutrophils and immature DCs [57,58]. In cancer, TLR5 has been shown to be highly expressed in patients with gastric carcinoma and breast cancer [59,60]. In colon cancer, TLR5 knockdown enhanced tumor growth and inhibited tumor necrosis in a xenograft mouse model of CRC [61]. Treatment of CRC mice with the TLR5 ligand flagellin induced infiltration of neutrophils. This exerted an anti-tumor effect, resulting in increased necrosis and decreased tumor growth [61]. Similarly, in breast cancer, TLR5 activation in vitro inhibited tumor cell proliferation and colony formation by decreasing cyclin B1, cyclin D1 and cyclin E2 expression and increasing CDK inhibitor p27. In vivo, the same study showed that treatment with the TLR5 ligand flagellin reduced tumor volume, increased tumor necrosis and increased leukocyte infiltration [59]. A subsequent study in breast cancer cells showed that flagellin induced autophagy by activating MAP1 S, which regulates the TLR5 signaling through enhancement of NF-κB activity and cytokine secretion [62]. The study showed that knockdown of MAP1S abrogated the suppression of tumor growth and migration caused by flagellin treatment [62]. In addition to breast, gastric and colorectal cancers, TLR5 was shown to be expressed and functionally active in prostate cancer cell lines [63]. Stimulation of prostate cancer cells with flagellin induced the secretion of chemokines responsible for the attraction of macrophages, CTLs and NK cells [63]. 

### 2.5. TLR7 and TLR8—Primarily Anti-Tumoral 

TLR7 and TLR8 are structurally related members of the TLR family that can be stimulated by ssRNA [64,65]. TLR7 is mainly expressed in plasmacytoid DCs and B cells, whereas TLR8 is primarily expressed in monocytes, macrophages and myeloid DCs. In cancer, TLR7/8 have been shown to be expressed in lung cancer, colorectal cancer and PDAC [66,67]. Since the activation of TLR7/8 triggers induction of a Th1-type innate immune response, their agonists have been suggested to be a promising strategy for cancer treatment. The small molecule agonist, R848, was shown to decrease PD-1 expression on T cells and enhance antigen-specific CD8 cytotoxicity in a sarcoma mouse model [68]. In pancreatic ductal adenocarcinoma (PDAC), R848 was shown to primarily act on stromal cell TLR7 rather than tumor cells and induce CD8 T-cell infiltration, activation and cytotoxicity, and decrease Treg cells [69]. In a lung cancer mouse model, TLR7 stimulation with R848 induced DC activation, increased CD8+ T and NK cells and decreased Treg cells [70]. R848 also reduced tumor growth compared to control, but did not show any effect on proliferation or apoptosis of LLCI cells in vitro [70]. Another study showed that R848 decreased MDSCs and decreased their immunosuppressive capacity on T cell proliferation in the CT26 colon carcinoma animal model [71]. This resulted in decreased tumor growth in R848-treated mice compared to control. Similar observations were reported for TLR8 ligands such as Poly-G3 and ssRNA40 [72]. TLR8 activation using these ligands was shown to block effector T cells from entering senescence by inhibiting tumor-derived endogenous cyclic adenosine monophosphate (cAMP), which is responsible for this conversion. This resulted in a better anti-tumor immune response and decreased tumor growth in a melanoma mouse model [72].

Despite these positive findings supporting the use of TLR7/8 agonist-based therapies in cancer, two studies showed opposing results. Grimmig et al. developed TLR7 and TLR8 overexpressing PANC1 cell lines, and showed that tumor growth in vivo of these cells was enhanced when compared with empty vector PANC1 cells [66]. Stimulation of these cells with R848 induced an increase in gene expression levels of NF-κB and COX-2 compared with untreated cells, resulting in chemoresistance [66]. The study did not analyze the effect on immune cells in the TME. Similarly, Cherfils-Vicini et al. showed that stimulation of TLR7 and TLR8 receptors in lung A549 cells induced activation of NF-κB and increased cell survival and resistance to chemotherapy [67]. These two studies showed that TLR7/8 agonists activated tumor-intrinsic mechanisms related to survival and treatment resistance. This contrasts with the studies detailed above, which showed immune-related mechanisms for TLR7/8 ligation in cancer. 

### 2.6. TLR9—Pro-Tumoral and Anti-Tumoral—Action Mediated through APCs

TLR9 is expressed in intracellular compartments of pDCs, monocytes, B lymphocytes, T lymphocytes (CD4+ and CD8+), endothelial cells, keratinocytes and melanocytes. It is also expressed on the surface of tonsil and peripheral B lymphocytes, splenic DCs, gastric, intestinal and cervical epithelial cells [73]. The role of TLR9 in tumor progression and response to treatment varies considerably in the literature. Its role appears to be tumor-type dependent. In melanoma, renal cell cancer and colon cancer, TLR9 ligation has been shown to be protective. Intra-tumoral administration of the TLR9 agonist CpG-B into the skin of early-stage melanoma patients showed improved APC activation and recruitment of cDC1 and CD14+ APC to the injection site and draining lymph nodes [74]. This also resulted in increased expression of type I IFN genes and T cell priming. Similarly, intratumoral injection of CpG-B in melanoma and thymoma tumors had a suppressive effect on tumor growth, which is mediated through antigen presentation by cDCs and priming of CTLs [75]. In the murine colon tumor 26 (CT-26) model, intratumoral injection of CpG ODN resulted in a significant decrease in tumor growth and improved survival, but no mechanistic data was provided to associate the response with immune cells in the TME [76]. In contrast to these studies, TLR9 activation in PDAC has been controversial. This could be due to the high stromal involvement in PDAC, and which could vary considerably between mouse models. In a human orthotopic PDAC xenograft model, treatment with the TLR9 ligand CpG-ODNs after chemotherapy induced a longer median survival time compared to treatment alone or control [77]. More recently, Okada H et al. used a second generation TLR9 agonist designated as K3-SPG, and showed that intratumoral injection of the agonist suppressed tumor growth in animal models of PDAC and colorectal cancer [78]. The study further demonstrated the response to be associated with systemic anti-tumor effects and the generation of immunologic memory (through rechallenge of cured mice). In contrast, in models of early stage PDAC development (dysplastic and neoplastic pancreata), Zambirinis CP et al. showed that TLR9 ligation with CpG promoted the generation of an immunosuppressive TME by increasing the proportion of Treg cells and inducing proliferation of MDSCs [79]. The study further showed that TLR9 ligation accelerates pancreatic oncogenesis in a genetically engineered mouse model of PDAC, while silencing TLR9 slows oncogenesis and improves survival. 

## 3. The Effects of Radiation on TLRs

The majority of TLRs are activated by microbial ligands. However, a wide variety of molecules of endogenous origin have also been reported to engage TLRs and activate them in the absence of microbial challenge. This includes small and large RNAs, DNA, ATP, UTP, chromatin, histones, mitochondrial DNA, heat shock proteins and calreticulin [80,81,82,83]. Despite the scarcity of basic studies investigating radiation and TLRs in cancer, radiation is recognized as either a direct stimulant of TLR pathways, or indirectly through the damage it causes to target cells that subsequently activate TLRs. Curtin JF et al. showed that 20 Gy irradiation of GL26 (glioma), LLc1 (lung carcinoma), GL261 (glioma) and B16-F10 (melanoma) cells increases HMGB1 expression [84]. The authors showed that HMGB1 is an endogenous agonist for TLR2, which is released from dying tumor cells in response to radiation or other stress-inducing agents (e.g., temozolomide). Treatment of syngeneic intracranial gliomas using a combination of FMS-like tyrosine kinase 3 ligand (Flt3L) and conditional cytotoxic gene thymidine kinase (TK) resulted in significant tumor regression. The effect was mediated through the MyD88/TLR2/NFκB pathway, and resulted in the infiltration of DCs into the brain parenchyma and the activation of cytotoxic CD8 T cells. By blocking HMGB1 activity, Flt3L/TK-induced brain tumor regression was inhibited. However, the authors did not perform similar experiments using RT alone or in combination with Flt3L or TK. Given their published data, it is reasonable to hypothesize that tumor-derived HMGB1 induced by RT elicits endogenous TLR2 signaling and initiate a CD8+ T cell-dependent immune response. In contrast to TLR2, TLR1 activation by RT has been shown to have tumor-promoting effects. Ryu et al. showed that macrophages exposed to irradiated tumor cells (CT26 cell line) had increased iNOS activity, increased nitric oxide (NO) production and increased arginase activity [85]. Knockdown of TLR1 decreased iNOS and NO activity, while TLR1 overexpression had the opposite effect. The authors fell short of testing the combination of TLR1 inhibition with RT. 

In addition to HMGB1, an important mediator of TLR activity is p53. Shatz M et al. studied the dependency of TLR expression on the induction of p53 in response to radiation (among other stress-inducing agents). They showed that 10 Gy irradiation of cancer cell lines (U2OS, A549, HEPG2 and MCF7) increased the expression of TLR2, TLR3, TLR4, TLR6 and TLR9. The expression of TLRs 2, 3 and 9 was observed to be p53-dependent, and introduction of p53 into p53 null cancer cell lines induced expression of these TLRs [86]. This effect was not investigated by the authors in vivo, and it remains to be seen how TLR signaling and tumor p53 status influences the response to RT. 

Another mediator of TLR signaling with relevance to radiation is IRAK2. IRAK2 is an immune-responsive protein kinase and a transducer of the IL1/TLR signaling cascade. In an analysis of radioresistant and radiosensitive isogenic oral squamous cell carcinoma cell lines, Yu CC et al. showed that IRAK2 expression is decreased in the radioresistant cell line at baseline and after radiation (4 Gy) [87]. In vivo, mice inoculated with the cell line overexpressing IRAK2 had significantly reduced tumor growth at baseline and in response to RT (50 Gy/10 fractions) compared to mice implanted with the parental radioresistant cell line (low IRAK2). The study also showed that patients with higher IRAK2 expression had a better recurrence-free survival rate. The effects of IRAK2 were shown to be mediated through apoptosis, as overexpression of IRAK2 increased apoptosis in response to radiation through cleavage of caspases 3 and 8 in the irradiated cells. Additional studies are warranted to investigate how IRAK2 is mediated by TLR signaling in response to radiation.

Based on the few studies to date on radiation and TLRs in cancer, it can be assumed that radiation-damaged cells release DAMPs which bind to TLRs and activate canonical inflammatory pathways. In particular, HMGB1 is a major component of dying cancer cells after radiation, and it can drive NF-κB and ROS production via activation of TLRs.

## 4. Combining Radiotherapy and TLR-Based Therapy

The wide tissue distribution of TLRs makes it difficult to determine whether an agonist or an antagonist will be most effective therapeutically. The type of activated TLR can determine the type of cytokine profile secreted, and this can modulate the TME in positive or negative ways. Based on our review of the literature, we poise that the most accepted division is that TLRs 3, 5, 7, 8 and 9 are anti-tumoral and thus require an agonist, while TLRs 1, 2 and 4 are pro-tumoral and thus require an antagonist. There are exceptions, as discussed above. In addition, despite the literature supporting this categorization, most TLR-based treatments in cancer are based on agonists, with very few studies integrating RT with TLR-antagonists. 

### 4.1. Radiotherapy and TLR Agonist-Based Therapies: TLR3, TLR7/8 and TLR9

There is a growing body of preclinical evidence that agonists targeting TLR3, TLR5, TLR7/8, or TLR9 in combination with RT may lead to enhanced anti-tumor immunity. Hammerich L et al. developed a strategy for treating NHL comprised of a TLR3 agonist (poly-ICLC) to activate DCs in combination with RT and Flt3L [88]. A significant accumulation of intratumoral, cross-presenting DCs was observed and resulted in an influx of tumor-specific CTLs. The addition of anti-PD-1/PD-L1 immunotherapy increased the durable remission rate in mice and caused regression of untreated distant metastases in patients. A similar strategy was used by Oba T et al. in triple-negative breast cancer [89]. The group used a combinatorial in situ regimen comprised of intratumoral administration of Flt3L, RT, and in situ TLR3/CD40 stimulations, followed by surgical resection. The treatment regimen resulted in a significant induction and activation of Batf3-dependent conventional type 1 DCs and an increased frequency of tumor-specific CD8+ T cells in primary and distant non-irradiated tumors. A less aggressive in vitro strategy employing cisplatin and poly (I:C) was used by Mikulandra M et al. [90]. Pre-exposure of HNSCC cells to cisplatin and poly (I:C) rendered them highly sensitive to radiation-induced apoptosis. The study showed that the effects were mediated via the induction of G2/M cell cycle arrest, which is the most radiosensitive phase of the cell cycle. In a similar in vivo study, Yoshida S et al. used a radioresistant murine model of Lewis lung carcinoma (LLC-OVA) and showed that pre-treatment of mice with poly (I:C) rendered the tumors sensitive to RT (single dose of 15 Gy) [91]. Activation of the TLR3 pathway was essential for the anti-tumor effects of poly (I:C), while TLR3+ Batf3 DCs and CD8+ T cells were indispensable for the therapeutic efficacy of poly (I:C). Clinically, the efficacy of poly (ICLC) was investigated in combination with RT and temozolomide in patients with GBM [92,93]. Both studies reported acceptable rates of toxicity with the use of poly (ICLC) in combination with RT. The findings also showed that the addition of the TLR3 agonist led to a survival advantage, although this was compared with historical data. Another phase I/II clinical trial investigating the efficacy of co-administration of poly (ICLC) and RT in patients with lymphoma recently completed accrual, and it will be of significant interest to see how this strategy fares out in the clinic (NCT01976585). Taken together, the data on TLR3 agonist-based treatment in combination with RT is encouraging and supports its further development.

Therapeutic interest in combining RT with TLR7/TLR8 agonists came about because of the inherent anti-tumoral activity of TLR7/TLR8 agonists. The TLR7 agonist imiquimod was shown to induce autophagic cell death when combined with RT in melanoma [94]. In vitro, the authors showed that imiquimod reduces proliferation of melanoma cells and increases sensitivity to radiation via the ROS-mediated ERK pathway. Combination of the two treatments in a mouse model of melanoma increased autophagy and decreased tumor growth compared to control and imiquimod alone. This was shown to be a result of increased infiltration of cytotoxic CD4+ and CD8+ T cells, while Tregs and MDSC populations were decreased [94]. In GI tumors, both TLR7 and TLR8 agonists in combination with RT showed a significant decrease in tumor growth [95]. Demaria S. et al. showed that imiquimod acts as an adjuvant for RT-elicited in situ vaccination against breast cancer [96]. Similar data showing that the combination of TLR7/8 ligands with RT improves local and distant tumor control has been reported in murine tumor models of GI cancer, lymphoma, fibrosarcoma, lung and pancreatic cancer [71,95,97]. Mechanistically, TLR7/8 agonists were shown to enhance maturation of MDSCs and/or M2 macrophages, promoting their conversion to APCs and M1-like macrophages and resulting in stimulation of tumor-specific T cell responses [97]. A recent technological development by Zhang Y et al. allowed for the development of a peptide hydrogel that contains TLR7/8 agonists and which was used to polarize TAMs for overcoming radioresistance [98]. Combined treatment of B16 melanoma tumors with the hydrogel and RT (8.1 Gy in 3 fractions) resulted in repolarization of TAMs to M1 macrophages, increased CD4+ and CD8+ CTLs, decreased Tregs and sensitized tumors to anti-PD-1 immunotherapy. Another group designed a nanoparticle comprised of the hydrophilic PD-L1 antagonist, PPA-1, co-assembled with the hydrophobic TLR 7/8 agonist, R848 [99]. When combined with RT, the nanoparticle triggered apoptosis of tumor cells and promoted the maturation of DCs. The addition of anti-PD-1/PD-L1 immunotherapy increased CTLs and further amplified the anti-tumor immune response. Collectively, there is compelling pre-clinical evidence and technological advancements that support the combination of TLR7/8 agonists with RT. A phase I/II clinical trial, integrating RT and imiquimod, recently completed accrual in patients with metastatic breast cancer (NCT01421017). Another phase I/II trial is ongoing in patients with advanced or metastatic solid tumors undergoing RT or standard of care treatment, in combination with TransCon TLR7/8 agonist. Data are therefore anticipated in the near future on the safety and efficacy of RT with TLR 7/8 agonists.

TLR9 activation is similar to TLR3/ TLR7/TLR8 activation and results in increased expression levels of costimulatory molecules such as TRAIL, which can induce tumor cell death, and CCR7, which promotes trafficking of APCs to lymph nodes. TLR9 activation has been shown to promote tumor regression directly through the action of TRAIL, and indirectly through the activation of NK cell-mediated tumor killing. Zhang H et al. investigated the combination of a TLR9 agonist with RT in a murine xenograft model of Lewis lung carcinoma [100]. They showed that the addition of a TLR9 agonist to single dose RT (20 Gy) could activate CD8+ CTLs and increase the proportion of pDCs and NK DCs. These effects were associated with a significant decrease in tumor growth in the RT + TLR9 agonist group compared to either treatment alone. Mice in the R + TLR9 agonist treatment group also had significantly reduced lung metastases and improved survival compared to all other groups. Younes AI et al. investigated the effects of adding a TLR9 agonist (CpG-A ODN) delivered in a virus-like particle, in combination with RT murine models of metastatic lung cancer (344SQ) and colon carcinoma (CT26) [101]. The authors showed that RT (12 Gy × 3 fractions) significantly increased the percentages of pDCs as well as CD4+ and CD8+ CTLs within the tumor. These immune-stimulating effects were associated with significant tumor regression in the irradiated site and distant non-irradiated sites, indicating a tumor-specific immune response. Another similar study was performed in canine tumors, where SBRT was combined with agonists for TLR3, TLR9 and OX40 [102]. The addition of TLR3/TLR9/OX40 to SBRT resulted in local depletion of Tregs and TAMs, but it is difficult to ascertain if all three agonists were needed to achieve this effect. Mechanistically, Yuan et al. showed that TLR9 ligation enhances the radiosensitivity of lung cancer cell lines via the p53 pathway. The authors used a single cell line (A549), but showed compelling evidence that treatment with a TLR9 agonist increases the radiosensitivity of lung cancer cells. They further demonstrated that CpG ODN treatment results in activation of the p38 MAPK-p53- pathway, which significantly enhances radiation-induced apoptosis and increases the radiosensitivity of A549 cells [103]. The combination of TLR9 agonist CpG ODN treatment with RT downregulated PD-L1 expression in NSCLC H460 cells, compared to control and RT alone. In addition, expression of NF-κB p65 protein was significantly induced by radiation, but was dramatically downregulated by the combination treatment compared to control [104]. 

### 4.2. Radiotherapy and Antagonist-Based Therapies: TLR1, TLR2, TLR4

With a few notable exceptions, stimulation of TLR1, TLR2 and TLR4 often leads to tumor-promoting effects by inducing immunosuppressive and antiapoptotic signals. Most studies on TLR-based treatment and RT have involved agonists, as described above. Few studies investigated the inhibition of TLRs in combination with RT. Fengming Lan et al. showed that the TLR1 gene is a direct target of miR-15a/16 in NSCLC cell lines [105]. TLR1 blockade with miR-15a/16 in vitro and in vivo increased radiosensitivity of A549 NSCLC cells by inhibition of the NF-κB pathway. Their results showed that pre-treatment with miR-15a/16 reduced cell viability and increased apoptosis after radiation. Tumor volume and TLR1, BCL-2, Cyclin D1 and survivin protein levels were significantly reduced in the combined treatment group with miR-15a + RT or miR16 + RT compared to control or single modality treatment. In the case of TLR2, Grabowski M et al. developed a small-molecule antagonist (MMG-11) against TLR2 [106]. MMG-11 was shown to inhibit ligand-induced interaction of TLR2 with MyD88 and reduce MAP kinase and NF-κB activation. The use of MMG-11 could provide promising data in cases where RT activation of TLR2 promotes tumor progression and/or metastasis [107]. To date, there are no studies that have integrated a TLR2 inhibitor with RT. Similar to TLR2, TLR4 inhibition has been shown to have antitumor properties. Currently, there are two commercially available inhibitors of TLR4. Kashani B et al. used the small molecule inhibitor of TLR4 (TAK-242—Resatorvid) in mouse models of ovarian and breast cancer, and found that TAK-242 halted cancer cell proliferation by inducing cell cycle arrest and apoptosis through the modulation of genes involved in these processes [108]. Using a mouse model of colon cancer, Pastille E. et al. showed that blocking TLR4 signaling by TAK-242 during the inflammatory phase of CAC strongly diminished the development and progression of colonic tumors. This was accompanied by decreased numbers of infiltrating macrophages, and reduced levels of pro-inflammatory cytokine in the colon compared to CAC control mice [109,110]. Despite these encouraging data and the availability of a small-molecule TLR4 inhibitor, there are no published reports on the combination of RT and TLR4 inhibition in solid tumors.

## 5. Conclusions

Radiotherapy is a powerful tool with more than just DNA-damaging capability. RT is now well established to have immune-modulating properties that can have antitumoral and pro-tumoral properties. Such effects depend on a host of factors such as the radiation dose, fractionation and type of tumor. An understanding of how RT manifests its effects on a particular tumor is the first step in deciding how TLR-based therapy might further enhance RT’s tumor-damaging properties, or limit its tumor-promoting properties. Liu et al. developed a gene signature that is based on TLR and TLR-related genes which can predict a chemotherapy response in HCC [111]. A similar gene signature for RT is an important next step. At the time of writing this review, a total of 20 clinical trials in cancer included a TLR target. Fifteen of those trials included RT in addition to TLR-based therapy. TLR ligands as monotherapies can have limited efficiency in cancer, but their combination with RT can have potent synergistic effects that curb not only local tumor growth, but also the growth of distant metastases.

## Figures and Tables

**Table 1:** Summary of pro and anti-tumoral effects of TLR ligation in cancer. The number of studies for each cancer site is provided in brackets.

Receptor	Ligand	Pro-Tumoral Effect (# of Studies)	Anti-Tumoral Effect (# of Studies)	Contributing Factors
TLR1/2	BLP	Colorectal (2); Gastric (1); Ovarain (1)	Lung (3); Melanoma (1)	Effects are modulated through cancer-intrinsic mechanisms which vary depending on tumor type
TLR3	Poly (I:C)	Esophageal (1); Breast (1); Gastric (1); HNSCC (1)	Lung (6); HNSCC (2); HCC (5); CRC (2); Neuroblastoma (1); Clear cell carcinoma (1); Breast (1); Cervical (1); Prostate (1); Melanoma (1)	Primarily anti-tumorigenic effects that are cancer-intrinsic and/or involve dendritic cells and CD8 T cells. Few correlative and in vitro studies suggest a pro-tumoral effect.
TLR4	LPS	Lung (3); Breast (5); PDAC (1)	-	Pro-tumorigenic effect that is primarily cancer-cell intrinsic with limited immune involvement.
TLR5	Flagellin	-	Gastric (1); CRC (1); Breast (3); Prostate (1)	Anti-tumorigenic effect mediated through neutrophils (likely N1).
TLR7/8	ssRNA	PDAC (1)	Sarcoma (1); PDAC (1); Lung (1); CRC (1); Melanoma (1)	Anti-tumorigenic effect mediated through NK and CD8 T cells and decreasing MDSCs and Tregs
TLR9	CpG	PDAC (1)	Melanoma (2); CRC (1); PDAC (2)	Mainly anti-tumorigenic effects mediated through recruitment of cDCs and priming of CTLs

## Data Availability

Not applicable.
